# Regulation of Porcine Plasmacytoid Dendritic Cells by Cytokines

**DOI:** 10.1371/journal.pone.0060893

**Published:** 2013-04-08

**Authors:** Nils Lannes, Artur Summerfield

**Affiliations:** Institute of Virology and Immunoprophylaxis, Mittelhäusern, Switzerland; Kantonal Hospital St. Gallen, Switzerland

## Abstract

Plasmacytoid dendritic cells (pDC) are the most potent producers of type-I interferon (IFN) and represent the main interferon (IFN)-α source in response to many viruses. Considering the important roles played by type I IFN’s, not only as antiviral effectors but also as potent alarming cytokine of the immune system, we investigated how such responses are regulated by various cytokines. To this end, we stimulated enriched pDC in the presence or absence of particular cytokines with a strong activator, CpG DNA, or a weak activator of pDC, foot-and-mouth disease virus (FMDV). Alternatively, we pre-incubated pDC for 16 h before stimulation. The pro-inflammatory cytokines tested Interleukin (IL)-6, IL17A, tumour necrosis factor (TNF)-α did not influence IFN-α responses except TNF-α, which promoted responses induced by FMDV. The haematopoietic cytokines Fms-related tyrosine kinase 3 ligand (Flt3-L) and granulocyte-macrophage colony-stimulating factor (GM-CSF) had enhancing effects on pDC activation at least in one of the protocols tested. IFN-β and IFN-γ were the most potent at enhancing FMDV-induced IFN-α, up to 10-fold. Interestingly, also the Th2 cytokine IL-4 was an efficient promoter of pDC activity, while IL-10 was the only negative regulator of IFN-α in pDC identified. The cytokines enhancing IFN-α responses also promoted pDC survival in cell culture with the exception of GM-CSF. Taken together this work illustrates how the cytokine network can influence pDC activation, a knowledge of relevance for improving vaccines and therapeutic interventions during virus infections, cancers and autoimmune diseases in which pDC play a role.

## Introduction

Plasmacytoid dendritic cells (pDC) represent a major source of interferon (IFN)-α/β and are specialized in sensing viruses. They represent 0.1–0.5% of porcine peripheral blood mononuclear cells (PBMC) [1]. Besides their ability to secrete high amount of type-I IFN, pDC can function as antigen presenting cells, promote immunity or alternatively mediate tolerance [2].

Due to the sensitivity of foot and mouth disease virus (FMDV) to type-I IFN *in vitro* [3] and *in vivo* [4], pDC may play an important role in early immunity against FMDV. In fact, it has been demonstrated that *in vivo* IFN-α responses induced during infection of cattle by FMDV are mediated by pDC [5]. However, FMDV, like other non-enveloped viruses, do not efficiently trigger pDC activation, at least *in vitro* [6], [7].

For human pDC, it has been demonstrated that the level of IFN-α production by pDC is controlled by distinct cytokines [8]. We therefore hypothesized that cytokines could promote the weak pDC responses to FMDV and aimed to characterize the impact of several cytokines secreted by T helper, myeloid and stromal cells on IFN-α responses and pDC survival. Stimulatory effects were found with haematopoietic cytokines, Th1 and Th2 cytokines, type I IFN and only one of the analysed pro-inflammatory cytokines. Anti-inflammatory interleukin (IL)-10 was the only suppressive cytokine identified.

## Materials and Methods

### Ethics Statement

Bleeding and care of donor pigs was carried out following ethics approval of the animal licence BE26/11 provided by the Canton of Bern, Switzerland.

### Enrichment and Detection of pDC Population

Peripheral blood mononuclear cells (PBMC) were isolated from citrated blood of specific pathogen-free pigs kept at our institute using Ficoll Paque (1.077 g/L, Amersham Pharmacia Biotech AG, Dubendorf, Switzerland) density centrifugation [9]. For enrichment of pDC, CD172a^+^ cells were sorted using either monoclonal antibodies (mAb) 74-22-15A (ATCC, LGC-Promochem, Molsheim, France) or mAb 74-22-15 (hybridoma kindly provided by Dr. A. Saalmüller, Veterinary University, Vienna, Austria), and a magnetic sorting system (MACS; Miltenyi Biotech GmbH, Bergisch-Gladbach, Germany). Following enrichment, pDC, identified as CD4^high^CD172a^low^ cells by flow cytometry [10], represented 2–5% of all cells. For CD4, mAb PT90A (VMRD, Pullman WA; now available from Washington State University, Pullman, WA, USA) was employed.

### Culture of Cells

Baby Hamster Kidney (BHK) 21 cells were grown in Glasgow's minimum essential medium (GMEM, Life Technologies) supplemented with 5% v/v foetal bovine serum (FBS, Biowest, Nuaillé, France) at 37°C, 6% CO_2_. For the production of virus, the cells were cultured in serum-free conditions. CD172a^+^ cells were cultured in Dulbecco's modified Eagle's minimal essential medium (DMEM) plus GlutaMAX™-I (GIBCO, Life Technologies, Basel, Switzerland) supplemented with 20 µM of β-mercaptoethanol (Life Technologies) at 39°C and 6% CO_2_.

### Production of Virus

FMDV O UKG 2001 was propagated in BHK-21 cells [11]. In order to avoid heparin-sulfate adaptation of FMDV, the virus was used with a maximum of three passages in BHK-21 cells after isolation from pigs. The viral titres were determined as described [12]. Mock antigen was prepared from uninfected BHK-21 cells in the same manner as FMDV.

### Porcine Recombinant Cytokines

The following bioactive porcine cytokines were added to the culture of CD172a^+^-sorted cells: tumour necrosis factor-α (TNF-α), granulocyte macrophage colony stimulating factor (GM-CSF), fms-like tyrosine kinase receptor-3 ligand (Flt3-L), IL-2, IL-4, IL-6, IFN-α, IFN-β, IFN-γ, IL-10 and IL-17A. The following cytokines were prepared in house: TNF-α [13], GM-CSF [14], Flt3-L [15], IL-4 [16], IFN-α [17] and IFN-β [18] using transient expression in HEK293 cells. IL-2 was kindly provided by Dr. Shigeki Inumaru (National Institute of Animal Health, Ibaraki, Japan). IL-6 and IL-17A were purchased from Kingfisher Biotech Inc. (St Paul, MN, USA). IFN-γ and IL-10 were purchased from R&D Systems (Abingdon, UK). Data on biological activity of the commercial cytokines are published on the webpage of the suppliers.

### Stimulation of Enriched pDC

Freshly isolated CD172a^+^ cells (4×10^6^cells/ml) were stimulated in 100 µl of serum-free medium with FMDV at a multiplicity of infection (MOI) of 5 tissue culture infectious dose_50_ (TCID_50_)/cell or 10 µg/ml of CpG D32 (Eurofins, Ebersberg, Germany), in absence or in presence of cytokines. Cytokines were also used in combinations. Supernatants were harvested after 24 h and IFN-α was detected by ELISA [19]. In some experiments, the cells were stimulated 16 h after pre-incubation with the cytokines.

### Determination of Apoptosis and Survival of Cells

CD172a^+^ cells were cultured and stimulated as described, harvested after 16 h or 24 h at 39°C, labelled with AnnexinV- fluorescein isothiocyanate (FITC) (Bender MedSystems, Vienna, Austria), CD172a and CD4 followed by isotype-specific R-phycoerythrin (R-PE) and FITC conjugates (Southern Biotechnology Associates, Birmingham, AL, USA). Cells were analyzed using three-colour flow cytometry. pDC and monocytes/conventional DC (cDC) were gated based on their CD172a/CD4 phenotype. The absolute number of surviving cells was determined by employment of CountBright counting beads (Life Technologies) following the manufacturers instruction, combined with a forward/side scatter gate to exclude cell debris and shrunken dead cells.

### Statistical Analysis

Significant differences were determined with SigmaPlot v11 using the Mann-Withney Rank Sum Test for the IFN-α responses and the t-test for the survival. Both tests were performed with a cut-off p-value <0.05.

## Results

### Cytokines Modulate the Level of IFN-α Produced by Enriched pDC

**Figure 1:**
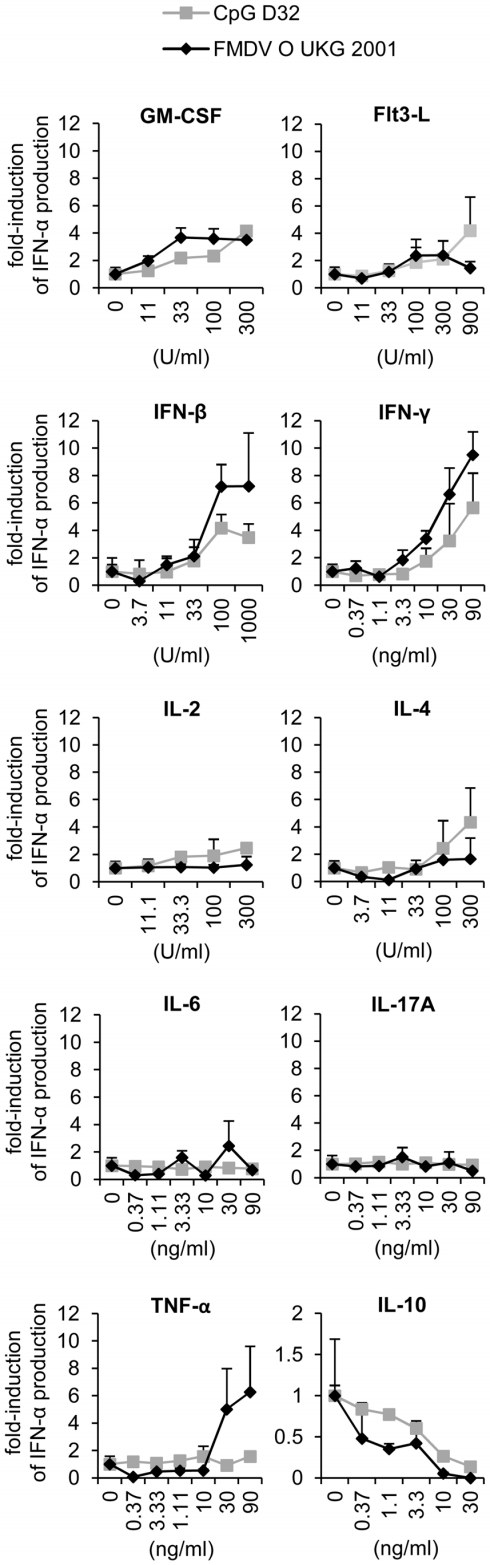
Cytokines influence the level of IFN-α production by enriched pDC in a concentration dependent manner. IFN-α responses of CD172a^+^-sorted cells stimulated with CpG (10 µg/ml; grey line) or FMDV (MOI of 5 TCID_50_/cell; black line) for 24 h in presence of cytokines at indicated concentration. After stimulation, supernatants were collected and IFN-α was measured by ELISA. The marker represents the mean value of 2 independent experiments with each condition performed in triplicate cultures. The error bar represents the standard deviation.

The first aim was to identify cytokines influencing pDC activation and to determine the dose-response relationship of their effects (Figure 1). From the pro-inflammatory cytokines, TNF-α was the only cytokine which enhanced IFN-α, while IL-6 and IL-17A had no effect. However, only responses to FMDV were enhanced by TNF-α. The hematopoietic factors GM-CSF and Flt3-L, promoted IFN-α secretion. Interestingly, both classical Th1 and Th2 cytokines, IFN-γ and IL-4 respectively, also had a positive effect on IFN-α secretion. Finally, only the anti-inflammatory cytokine IL-10 was inhibitory for pDC–derived IFN-α production (Figure 1). Our results also demonstrate that relatively high cytokine concentrations of at least 10 ng/ml or 100 U/ml were required to influence IFN-α responses by pDC.

**Figure 2:**
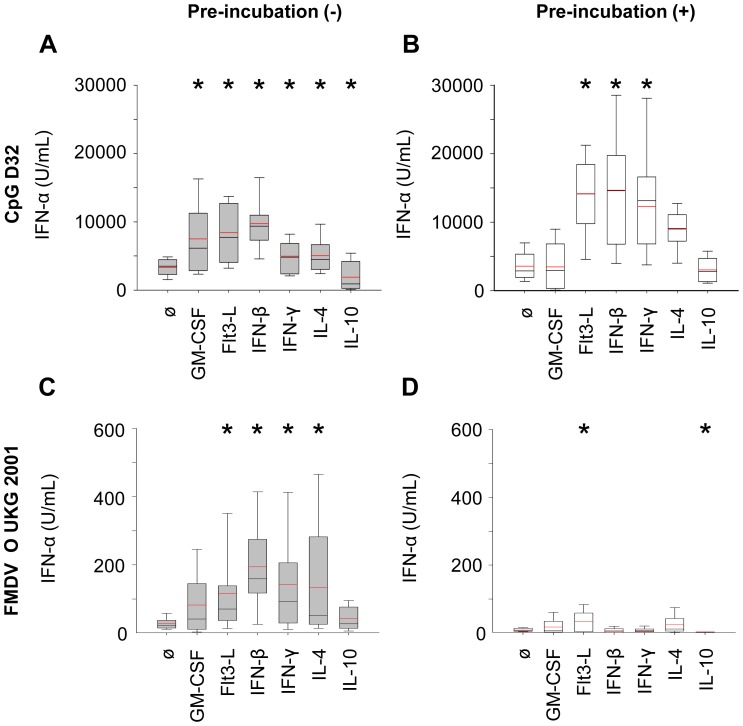
Boxplots showing the statistical analysis for the influence of cytokines on IFN-α production induced by CpG or FMDV in enriched pDC. Cells were cultured in presence of GM-CSF (100 U/ml), Flt3-L (100 U/ml), IFN-β (100 U/ml), IFN-γ (10 ng/ml), IL-4 (100 U/ml) or IL-10 (10 ng/ml), and either directly stimulated for 24 h (grey bars, A, C) or pre-incubated for 16 h before stimulation with CpG or FMDV for another 24 h (white bars, B, D). IFN-α in the supernatants was measured by ELISA. Values are shown as box plots representing at least 3 independent experiments with each condition performed in triplicate cultures. The black line represents the median value and the red line the mean value. The error bars represent the standard deviation. The asterisk indicates statistical significance between non-treated and treated cells (Mann-Withney Rank Sum test, P<0.05).

We further evaluated selected cytokines using concentrations of 10 ng/ml or 100 U/ml and stimulation of at least three independent cell preparations from 3 different pigs. As illustrated in Figure 2A and 2C, again, Flt3-L, IL-4, IFN-β and IFN-γ promoted a potent enhancement of the level of IFN-α-produced by enriched pDC stimulated with both CpG and FMDV. GM-CSF enhanced and IL-10 inhibited the IFN-α production upon stimulation with CpG but not FMDV. Nevertheless, considering the very low responses induced by FMDV stimulation, inhibitory effects of cytokines are difficult to measure in this model.

### Pre-incubation Modifies the Influence of Cytokines on IFN-α Responses of pDC

With the idea to have an even stronger effect of cytokines on pDC, we tested the impact of pre-incubation of the enriched pDC with the selected cytokines 16 h prior to their stimulation by CpG or FMDV. Surprisingly, when cells were pre-incubated in medium without cytokine and subsequently stimulated, similar levels of IFN-α as freshly stimulated cells were observed in response to CpG (Figure 2A and 2B). However, the treatment resulted in unresponsiveness to FMDV (Figure 2C and 2D). Using this protocol, Flt3-L was the only cytokine able to enhance the level of IFN-α production upon both CpG and FMDV stimulation. IFN-β and IFN-γ increased the level of IFN-α production of CpG-stimulated cells but not of FMDV-stimulated cells. IL-10 inhibited FMDV but not CpG-stimulated cells responses (Figure 2B and 2D).

### Synergistic Effects of IFN’s and Other Cytokines on IFN-α Responses

**Figure 3:**
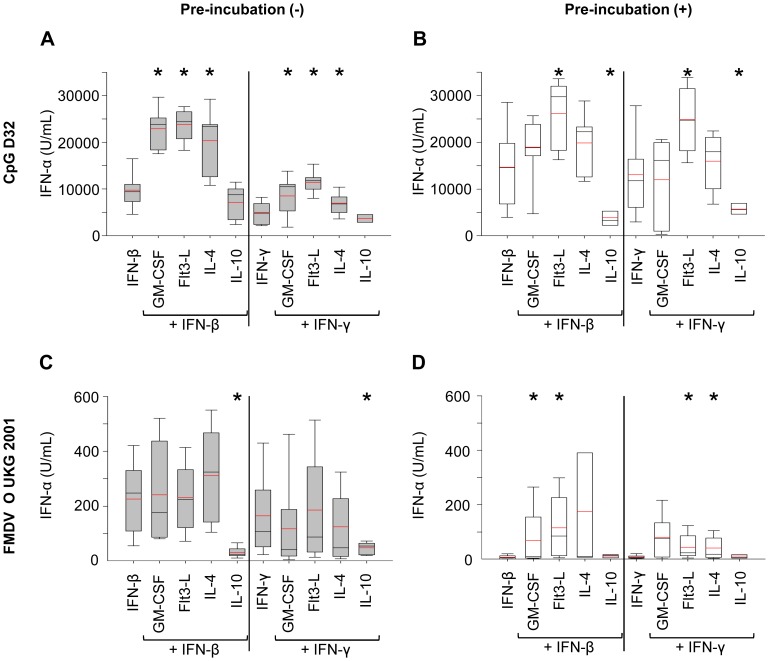
Synergistic effects of IFNs with other cytokines on IFN-α by enriched pDC. IFN-α responses of CD172a^+^-sorted cells stimulated with CpG or FMDV in presence of cytokine combined with type-I and II IFN are shown. The effects of IFN-β or IFN-γ in combination with GM-CSF, Flt3-L, IL-3, IL-4 or IL-10 using concentrations as in Figure 2 were tested. Cells were either directly stimulated for 24 h (grey bars, A, C) or pre-incubated for 16 h before stimulation with CpG or FMDV for another 24 h (white bars, B, D). After stimulation, supernatants were collected and IFN-α was measured by ELISA. Values are shown as box plots representing at least 3 independent experiments with each condition performed in triplicate cultures. The black line represents the median value and the red line the mean value. The error bars represent the standard deviation. The asterisk indicates statistical significance between non-treated and treated cells (Mann-Withney Rank Sum test, P<0.05).

Overall, the above results show that IFN-β and IFN-γ were among the most potent cytokines enhancing pDC activation. We were next interested to determine whether synergistic activities between the IFN’s and the other cytokines were identifiable. Combinations of IFN-β or IFN-γ with GM-CSF, Flt3-L, and IL-4 enhanced the level of IFN-α production by CD172a^+^ cells in response to CpG, when compared to the IFN’s alone (Figure 3A). Different results were obtained with FMDV-stimulated pDC for which no synergistic activities in terms of enhancement of pDC responses were identified. Nevertheless, combinations of both IFNs with IL-10 inhibited the IFN-α production compared to the corresponding IFN control (Figure 3C). In fact, IL-10 efficiently counteracted the influence of the promoting IFNs and reduced it to the level found with untreated cells stimulated with either CpG or FMDV (data not shown).

We next evaluated the impact of cytokines combinations when pre-incubated with the enriched pDC prior to stimulation. Combinations of IFN-β or IFN-γ with Flt3-L were able to enhance the IFN-α level of CpG-stimulated cells. In contrast, combinations of both IFNs with IL-10 inhibited the IFN-α production by CpG-stimulated cells. Combinations of both IFNs with GM-CSF did not enhance the IFN-α production upon CpG stimulation (Figure 3B). With FMDV, pre-incubation of the cells for 16 h before stimulation resulted in a lack of response or an overall lower IFN-α production. Only the IFN-β/Flt3-L, the IFN-β/GM-CSF, the IFN-γ/Flt3L and the IFN-γ/IL-4 combination enhanced the level of IFN-α production compared to IFN alone (Figure 3D).

### Apoptosis and Survival of pDC

**Figure 4:**
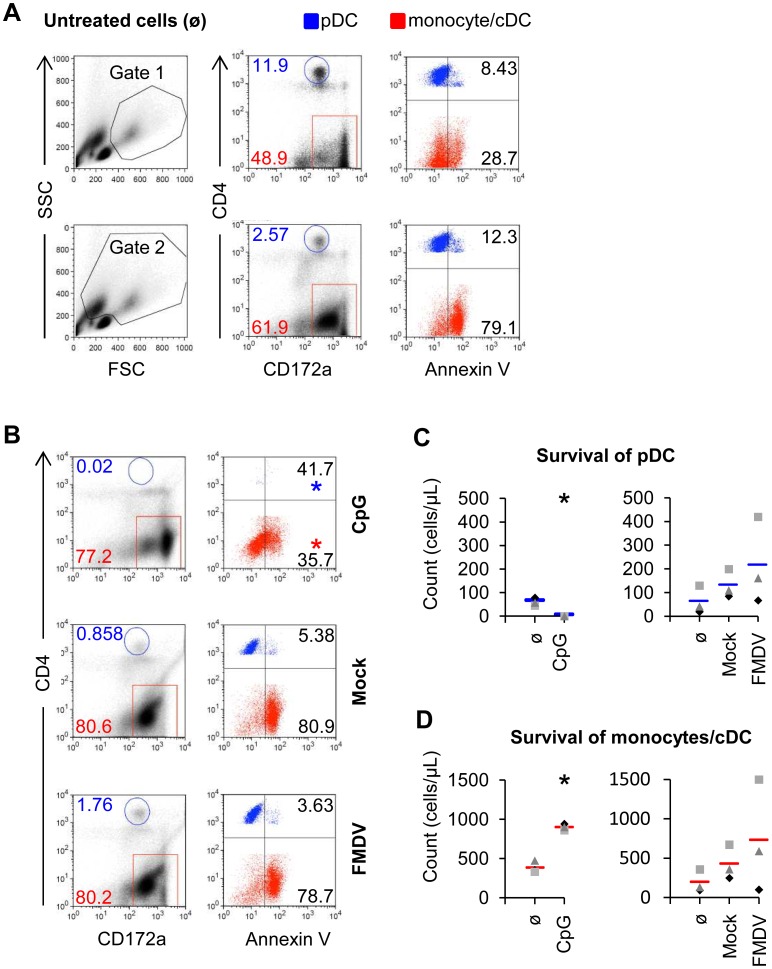
CpG but not FMDV deplete pDC population by inducing apoptosis. CD172a^+^ sorted cells were untreated (A) or stimulated with 10 µg/ml of CpG for 16 h or FMDV for 24 h (B, C, D). A. Three-color FCM of CD4, CD172a and AnnexinV, with a CD4/CD172a dot plot defining the pDC gate (CD4^high^CD172a^low^) and the monocyte/cDC gate (CD4^−^CD172a^+^) after culture in medium. On the left side plot, putative live cells and live and dead cells were defined according to the FSC/SSC profile, excluding lymphocytes and cell debris. On the right side plots, the AnnexinV expression of the gated pDC (blue) and monocytes/cDC (red) with percentages are shown. B. Apoptosis of pDC and monocytes/cDC after stimulation with CpG, Mock antigen and FMDV. On the left side plot, pDC and monocytes/cDC with percentages were gated on live/dead cells as described in A. On the right side plot, the AnnexinV expression of the gated cell populations with percentages is shown after CpG or FMDV stimulation. C, D. Absolute number of pDC and monocytes/cDC on gated live cells after culture of enriched pDC with CpG, FMDV or only medium. Each marker represents results from one individual experiment and the bar represents the mean value. The asterisk indicates statistical significance between treated and non-treated cells (t-test, P<0.05).

Considering the influence of cytokines on IFN-α response by pDC, we were interested to relate this to apoptosis and survival of pDC. As shown in Figure 4A, a gate on FSC^high^/SSC^high^ cells was combined with a gate on CD4^high^CD172a^+^ cells, to identify pDC and on CD4^−^CD172a^+^ cells for monocytes and cDC [1]. These populations did not contain many AnnexinV^+^ cells. However, after including the FSC^low^SSC^high^ cells in the gate, a high level of AnnexinV^+^ CD172a^+^CD4^−^ monocytes/cDC was found (Figure 4A). Backgating confirmed that the FSC^low^SSC^high^ contained the apoptotic monocytes/cDC, which had a reduced CD172a expression (data not shown). For the pDC defined as CD4^high^CD172a^+^ population only a minor fraction was identify as AnnexinV^+^. However, it appeared that apoptotic pDC have reduced CD4 and possibly also CD172a expression making their identification problematic. In fact, as shown in Fig. 4B, the CD4^high^CD172a^+^ population almost completely disappeared after CpG stimulation. We concluded that the most accurate way to determine survival was by assessing the absolute numbers of AnnexinV- cells among the FSC^high^/SSC^high^ cells in the phenotypic gates defined in Figure 4A. This analysis confirmed that CpG stimulation depleted pDC while enhancing the number of monocytes/cDC. FMDV neither influenced the survival of pDC nor monocytes/cDC in a statistically significant manner (Figure 4C and 4D).

**Figure 5:**
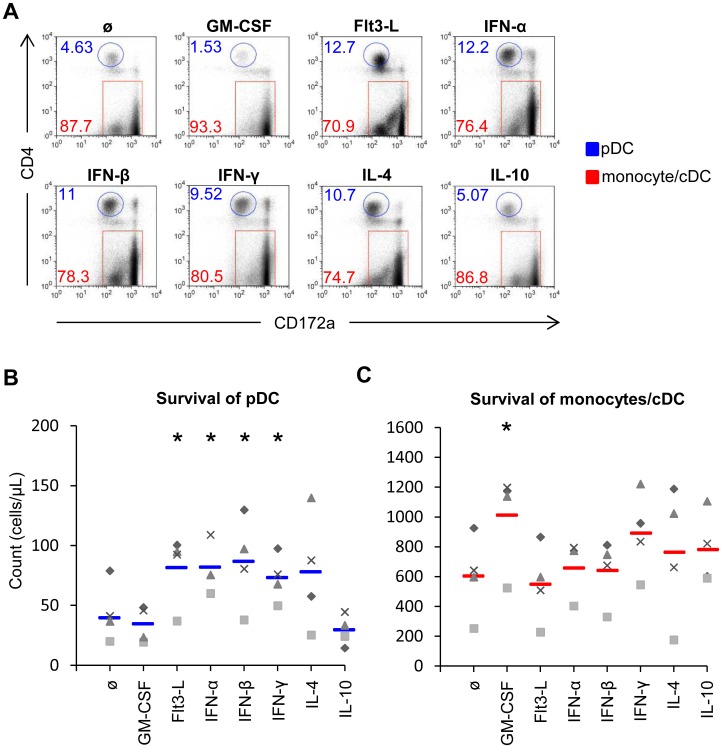
Impact of cytokines on pDC and monocytes survival. Enriched pDC were cultured in presence of the indicated cytokines (concentrations as defined in Figure 2) for 16 h at 39°C. In A, the frequencies of pDC (blue) and monocytes/cDC (red) are shown on gated live cells. The plots are of a representative animal. In B and C, the survival of pDC and monocytes/cDC was determined on live cells. Each marker represents one individual culture of on animal and the bar the mean value. The asterisk indicates statistical significance between treated and non-treated cells (t-test, P<0.05).

We next applied these analyses to determine the impact of cytokines on survival of pDC and monocytes. In Figure 5A, the CD172a/CD4 dot plots give a visual impression on the effects of the cytokines, while Figure 5B and C give the absolute numbers. Flt3-L, type I IFN and IL-4 induced the survival of pDC, and IFN-γ promoted both pDC and monocytes/cDC survival. GM-CSF only promoted the survival of monocytes/cDC (Figure 5B and 5C). Altogether, these results demonstrate that the cytokines enhancing IFN-α responses often but not always promote pDC survival.

## Discussion

The present study confirms and elaborates on the important impact of cytokines on pDC activation and survival. High level of IFN-α production did not always relate to the high number of surviving pDC indicating that other factors such as priming effects are mediating the effects. An example for this are the known effects of type I IFNs on the expression of IRF7 [20], a key transcription factor for IFN-α [21]. We also demonstrated that the effect of cytokines is not identical when CpG is compared to FMDV stimulation. This is not surprising, considering that cytokines may modulate pDC elements, which indirectly influence IFN-α responses such as uptake receptors or the antiviral status of the cells. For example, entry of FMDV requires specific integrins at the cell surface [22] while CpG utilizes a multilectine receptor, DEC-205 [23]. It is also possible that the differences are caused by the fact that pDC respond to CpG via toll-like receptor (TLR)9 [19] and FMDV via TLR7 [7]. Compared to CpG, FMDV is a very inefficient stimulator of pDC even in the presence of promoting cytokines. There are several possible explanations for this. First, the virus seems inefficient in attaching and entering pDC, and this can be improved by complexing it with specific antibodies which promote FcγRII-mediated uptake [6]. We know from previous work that pDC activation by FMDV is not influenced by cell culture adaptation to heparin sulphate receptors [7]. Second, after endocytosis of FMDV by pDC viral RNA might by delivered mostly to the cytosol. As FMDV cannot efficiently replicate in pDC, little RNA will be available for TLR7 triggering. Third, viral inhibitors of the IFN system such as L^pro^ [24] might prevent pDC activation. Future studies are required to address this issue but it appears that in vivo pDC are activated by FMDV, resulting in a transient early systemic IFN-α response [5], [25], indicating that pDC represent a relevant cell type during FMDV infection.

In general terms, our study support the concept that pDC are regulated at various steps of the innate and adaptive immune response by the cytokine network. The inflammatory cytokines tested were not able to promote activation of pDC with the exception of TNF-α, which enhanced the levels of IFN-α in response to FMDV but not CpG. A possible explanation for this could be antiviral effects controlling the level of viral proteins known to interfere with the IFN system such as L^pro^ of FMDV [24]. The observation that IL-17A did not influence pDC responses would relate to the fact that this cytokine is typically associated with bacterial infections [26] for which strong pDC responses are less relevant. It is also understandable that during strong inflammatory cytokine responses often associated with tissue damage an additional potentiation of IFN-α responses could be detrimental.

In contrast, the two hematopoietic cytokines tested (GM-CSF and Flt3L) were able to enhance pDC responses. Stromal cells are the major source of Flt3-L, which is the key cytokine in pDC development [27]. Its receptor (Flt3) is also expressed on differentiated porcine pDC [15] and this is related to the capacity of Flt3-L to enhance pDC activation and survival. Surprisingly, GM-CSF also promoted pDC activation (by CpG only) although our previous study demonstrated a lack of GM-CSF receptors on pDC [10]. A possible explanation could be that GM-CSF acts indirectly via promoting monocyte survival and activation, which in turn support pDC. This needs further clarification by using pure pDC populations in future studies.

Type-I IFNs possess antiviral activity and stimulate their own production allowing the release of high levels of IFN-α via a positive feed-back loop mechanism [28]. This is also functional in pDC [20], [21], [28]. Type I IFNs promote porcine pDC survival (this study) and also efficiently prime pDC by inducing high levels of IRF7 [20]. We think that for these reasons IFN-β was the most potent enhancer of IFN-α responses when all experimental conditions employed in the present study are considered.

The final group of cytokines investigated were classical Th1 and Th2 cytokines. This was considered important knowing the homing characteristics of pDC to the T-cell area in the activated lymph nodes [29]. Interestingly, both IFN-γ the major Th1 and IL-4 the major Th2 cytokine promoted pDC activation and survival. This indicates that the Th1/Th2 system does not imprint a particular bias on pDC but rather supports their function during a T-cell response. Nevertheless, it should be noted that porcine IL-4 has been reported to functionally differ from human and mouse IL-4 at least with respect to its ability to promote B-cell activation and this author has questioned the role of IL-4 in the porcine Th1-Th2 paradigm [30]. Furthermore, IL-4 has been difficult to detect, both at the protein and mRNA level, in vitro and in vivo [30]. It is possible that in particular breeds of pigs IL-13 could have a more prominent role for porcine Th2 responses [31]. On the other hand, both IL-4 and IL-13 are able to induce the generation of monocytes-derived DC in the pig [32] and to prevent apoptosis of endothelial cells [33]. Independent of this issue requiring further clarification, pDC are more likely to promote Th1 responses through secretion of type I IFNs, IL-12 and TNF-α [2]. We and others have also found this profile of cytokines for porcine pDC [1], [34].

As expected from studies with other species [8], the anti-inflammatory cytokine IL-10 represents a main negative regulator of porcine pDC. This cytokine is produced by regulatory T cells, and many other cells such as monocytes/macrophages, B cells and DC during the terminal phase of an immune response, where it has important anti-inflammatory functions to prevent unnecessary tissue damage caused by the immune system. Interestingly, pDC have been reported to control B-cell-derived IL-10 production [35], which together with the strong effects of IL-10 on pDC activation would fit into the concept of a vicious circle of chronic pDC activation such as described during systemic lupus erythemathosus [36]. Considering the necessity to regulate innate immune responses to avoid damaging effects of IFN-α during infections, it is not surprising that other cytokines such as include prostaglandin E2 and transforming growth factor-β [37]-[41] which counter-regulate pDC responses have been described. Their effect on porcine pDC was not addressed in the present study, but we expect a suppression of IFN-α responses as in other species.

Taken together, the knowledge on how cytokines regulate pDC responses can be employed to modulate the immune response towards a wanted direction. Considering the importance of type-I IFN as endogenous immunological adjuvant, cytokines could for instance be used to enhance pDC-derived type-I IFN during vaccination. Another area would be to employ such knowledge for immunotherapies of cancer, chronic virus infections and autoimmune diseases where pDC responses can be beneficial or unwanted.
